# Visuospatial working memory abilities in children analyzed by the bricks game task (BGT)

**DOI:** 10.1007/s00426-023-01803-1

**Published:** 2023-02-17

**Authors:** G. D’Aurizio, I. Di Pompeo, N. Passarello, E. Troisi Lopez, P. Sorrentino, G. Curcio, L. Mandolesi

**Affiliations:** 1 Department of Biotechnological and Applied Clinical Sciences, University of L’Aquila, Via Vetoio - Loc. Coppito, 67100 L’Aquila, Italy; 2 Department of Humanities, University of Naples Federico II, Naples, Italy; 3 Department of Motor Sciences and Wellness, University of Naples “Parthenope”, Naples, Italy; 4 Institut de Neurosciences Des Systemès, Aix-Marseille University, Marseille, France

## Abstract

The study of the development of visuospatial memory processes is useful for devising personalized educational interventions as well as for understanding the changes in cognitive functioning in an era characterized by technological progress. The present research is aimed at investigating spatial working memory ability in children that attended the first three years of primary school by means of the Brick Game Task (BGT), a novel visuospatial working memory test. BGT is a small-scale ecological test inspired by behavioral walking tasks with nine white bricks in different spatial configurations as well as to Corsi Block-Tapping test.

228 Italian children (121 *F*; mean age: 7.22 ± 1.18) were assigned to three groups based on the primary school class attended: Group 1 (*N* = 85; 40 *F*; mean age 6.18 ± .5), Group 2 (*N* = 61; 36 *F*; mean age 7.2 ± .83), and Group 3 (*N* = 82; 44 *F*; mean age 8.32 ± .94). All participants were asked to complete the Digit Span test, the Corsi Block-Tapping test, and to explore the three spatial configurations of the BGT with the form of Matrix, M-BGT, Cluster, CL-BGT, Cross, CR-BGT.

MANOVA revealed a main significant effect for Group (*F*_12,434_ = 15.06; *p* < .0001) indicating that the group of older obtained a better global executive performance than 1 and 2 groups. Multiple linear regression indicated that Corsi Block-Tapping test performance and Age significantly predicted the M-BGT score. Moreover, Corsi Block-Tapping test and Digit Span significantly predicted the CL-BGT performance, showing how a higher score results in a better CL- BGT performance. Finally, Corsi Block-Tapping test, Digit Span, and Age were positively associated with the CR- BGT performance. The present findings evidenced that novel BGT is a sensible visuospatial working memory task suggesting thus its use to assess the children’s executive performance in ecological way. These results open to the development of personalized educational interventions.

## Introduction

In light of recent technological advances, studying visuospatial working memory (VSWM) development in children is crucial to create personalized educational interventions. VSWM is a multiple-component cognitive system, comprised of a central executive and two sub-systems: the phonological loop and the visuospatial sketchpad. The phonological loop is involved in the processing and holding verbal information, while the visuospatial sketchpad is needed in processing and for holding visuospatial information (Baddeley, 2000, 2021). According to Logie’s multicomponent model (2003), the visuospatial sketchpad is divided into two major components: one for processing visual information (Visual Cache) and one for processing spatial information (Inner Scribe). Visual information can temporarily be stored in the former (such as color, shape, and static visual patterns) and movement sequences can be rehearsed in the latter (such as sequential locations and movements) and can be used in working memory for implementing planning and execution of movement.

Since VSWM skills involve the ability to remember shapes and colors, as well as their locations and movements, they facilitate letter/number recognition, reading, writing and math for young children (Fanari et al., 2019; Li & Geary, 2017). Moreover, they also predict adolescents' geometry achievement and general academic performance in early elementary school grades (Kyttälä & Lehto, 2008). Li and Geary (2013) analyzed the relationship between developmental growth in working memory systems and mathematics achievement from first to fifth grade. They found that developmental gains in visuospatial memory from first to fifth grades were associated with mathematics achievement in fifth grade, even after controlling for prior achievement, intelligence and other factors. According to Pham and Hasson (2014), visuospatial abilities also affect the development of reading skills. Through VSWM, children acquire the ability to recognize the orientation and shape of letters of the alphabet, as well as to associate those letters with the corresponding symbol of unknown words (Badian, 2005; Gathercole et al., 2003;).

The development of visuospatial abilities in children has been widely studied over the last decades, using a variety of methods and producing many reliable results (Isaacs & Vargha-Khadem, 1989; Palombi et al., 2022; Piccardi et al., 2013). Continuing to study this cognitive process is necessitated by society and technology evolution, and its impact on developmental trajectories. In addition, more researchers are stressing the need to develop more ecologic tools, to overcome the limitations of pen-and-paper testing in hospital and laboratory environment, possibly using computerized interfaces (Foti et al., 2020).

As clearly summarized by Buttelmann et al., (2020), the most common VSWM assessment tools, used with children, are span tasks. In these tasks, children are first exposed to an encoding phase (with the presentation of a sequence of stimuli) to subsequently go to a recall phase (when, after a short period of time, they have to repeat them in the correct or reversed order). VSWM span tasks show strong improvement in performance during childhood due to neuroanatomical and functional maturation of specific brain networks associated with executive functioning (Krogsrud et al., 2018; Mandolesi et al., 2009). While these well-known assessment tools offer the advantages of standardization, good reliability, and comprehensive normative data, they lack ecological validity. Increasingly, neuropsychology is concerned with understanding the relationship between assessment results and everyday performance (Spooner & Pachana, 2006)**.** Most of the current knowledge regarding the ecological validity of neuropsychological measures is the result of studies examining adult (Carretti et al., 2015) and older adult populations (Zarantonello et al., 2020). The relationship of test performance and everyday functioning in children has been less examined and is, therefore, less understood (Olson et al., 2013; Price et al., 2003). It remains a scientific priority to develop neuropsychological assessment tools that are ecologically valid in children, as such an approach would make it easier to draw conclusions about the functionality of children's adaptation and development. There have been several recent studies that have attempted to develop neurological assessment instruments with greater ecological validity, especially for the assessment of spatial abilities (Foti et al., 2020; Piccardi et al., 2014; Sorrentino et al., 2019). Despite such evidence, still the clinical and neuropsychological assessment is not characterized by ecological tools, that continue to represent a challenge for research in the context of development processes.

The aim of this study was to validate the Bricks Game Task (BGT), a small-scale ecological test inspired by behavioural walking tasks (with nine white bricks in different spatial configurations; Foti et al., 2011, 2015; Sorrentino et al., 2019) and the Corsi Block-Tapping test (Corsi, 1972). With respect to the Corsi test, the BGT offers the opportunity of investigating the cognitive and behavioural strategies used by a child to navigate in the peri-personal space. Using this new task, we are expecting children’s performance to be compared to that of the classical span tests (Corsi, 1972). We also expect different outcomes based on the group and spatial configuration of the BGT. As a first, we expect that groups with more schooling will perform better on the classic span task as well as the BGT. Furthermore, we expect that the Matrix configuration of the BGT will result in the easiest and thus children will perform the best, as compared to Cluster or Cross configurations, confirming some previous results research suggest (Foti et al., 2011, 2015; Sorrentino et al., 2019).

## Material and methods

### Participants

The whole sample was composed of 228 Italian children (121 females; mean age: 7.22 ± SD 1.18). Participants were assigned to three groups, based on which of the selected primary school class they attended: Group 1 (Gr1; *N* = 85; 40 *F*; mean age 6.18 ± SD 0.5), Group 2 (Gr2; *N* = 61; 36 *F*; mean age 7.2 ± SD 0.83), and Group 3 (Gr3; *N* = 82; 44 *F*; mean age 8.32 ± SD 0.94). Data were collected from two L’Aquila (Italy) primary public schools, before SARS-CoV2 pandemic. Exclusion criteria0 were: (a) presence of any neurological or neuropsychological deficits; (b) presence of any medical condition that might influence cognition performance; (c) diagnosis of learning disabilities. All participants had normal hearing and normal or corrected-to-normal vision.

Informed written consent to perform the task was obtained from the children’s parents. The study was approved by the Internal Review Board of the University of L’Aquila (n.16/2016) and was conducted in accordance with the 1964 Declaration of Helsinki.

### Bricks game task (BGT)

**Fig. 1:**
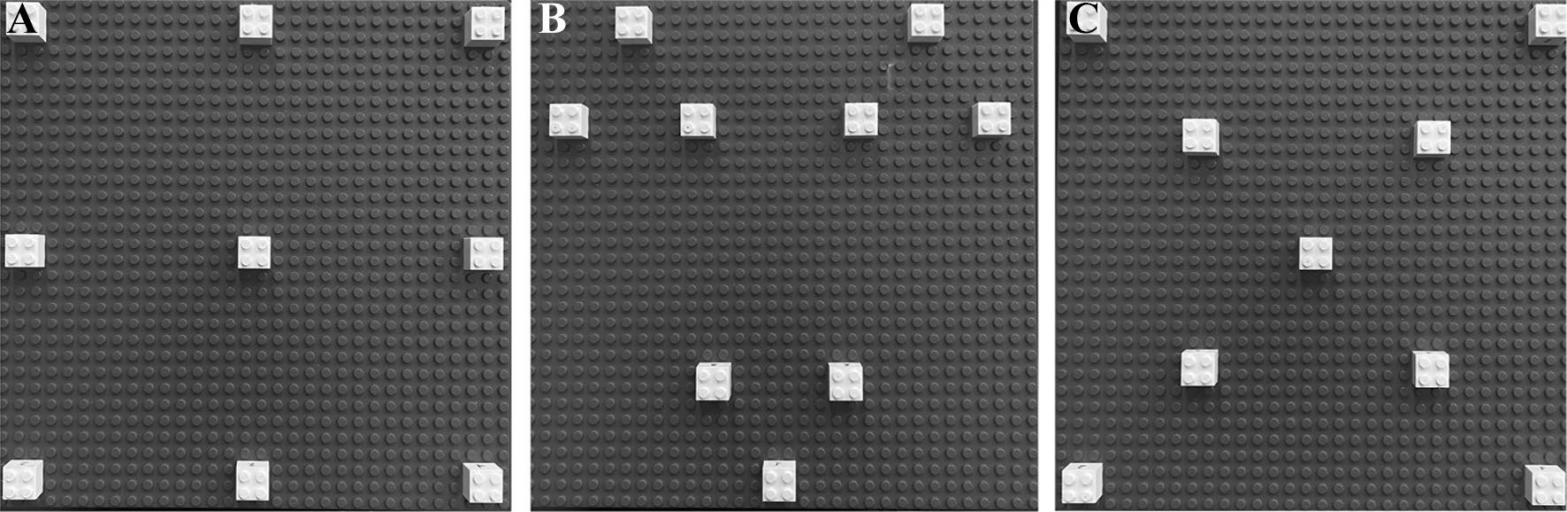
The Bricks Game Task. The three panels represent the Matrix **(A)**, Cluster **(B)**, and Cross configuration **(C)**, respectively

The Bricks Game Task (BGT) consists of a square blue platform (25 cm × 25 cm) in which nine white bricks (1 cm wide × 1 cm long × 2 cm high) are inserted and arranged with three different configurations: Matrix (M-BGT), Cluster (CL-BGT), and Cross (CR-BGT) (Fig. 1).

Being an ecological version of the Corsi Block-Tapping test (Corsi, 1972), that takes into account the characteristics of the peripersonal space, in BGT on the experimenter’s side the blocks are numbered for an easier identification. For each configuration presented in random way to participants, the experimenter taps a number of blocks at a rate of one block per 2 s using the index finger for tapping the blocks and lifting the hand straight up (for 2 s) before moving it to the next block. After this, the child has to tap the block sequence in the same order it was presented by the experimenter. The block sequences gradually increase in length and the score is the number of blocks in the longest sequence remembered correctly. This index is considered a BGT span measure. Three sequences are presented for every length (from 2 to nine blocks). If the participant correctly reproduces the three sequences, the test continues; otherwise, it is stopped, and the BGT span measure was recorded. Table 1 are shown the block sequences.

**Table 1:** Block sequences presented to children for each configuration

Sequences	Span	Sequences	Span
8 5 6 4 1 8	2	2 3 6 4 9 5 9 8 1 4 5 6 2 3 1 5 9 4	6
4 7 2 8 1 5 9 5 8	3	5 9 4 7 3 6 2 6 5 4 7 3 2 1 7 2 4 1 8 3 6	7
9 3 1 5 4 9 8 7 7 5 3 2	4	1 8 6 7 3 2 4 9 4 5 8 2 1 7 4 3 2 5 8 1 7 6 3 9	8
3 4 1 7 2 8 5 4 1 9 9 1 8 2 6	5	2 3 6 7 4 8 1 9 5 8 9 4 3 2 7 6 5 1 5 9 7 2 5 6 3 1 8	9

### Neuropsychological assessment

With the main aim to evaluate if the BGT could be a sensible visuospatial working memory task and then to use it to assess the children’s executive performance in ecological way, all children were evaluated in two classical working memory tasks.

*Digit Span task (DS;* Wechsler, 1991*).* This task requires the child to listen and correctly repeat a series of digits of increasing difficulty and length in the same order (DSF) or in the reverse (DSB) order of that read by the experimenter. The forward and backward DS conditions assess Attention functionality (Robertson et al., 1996), Working Memory (WM) capacity (Ramsay & Reynolds, 1995) and the sub-executive process as the ability to manipulate information in the mental workspace offered by WM and updating the information in WM.

*Corsi Block-Tapping test (CBT; *Corsi, 1972*).* The apparatus consists of 9 blocks fixed on the wooden structure. The examiner taps the blocks of a specific sequence that progressively increases in difficulty and length at a rate of one block per 2 s using the index. At the end, the participants must try to reproduce the sequence in the same order it was shown. CBT has been administrated to investigate individual differences in spatial skills and visuospatial memory (Berch et al., 1998). In both tasks, the span measure was recorded.

### Experimental procedure

All children were individually tested in a soundproof room of their own school, in which they performed firstly the digit span and the Corsi Block-Tapping tasks and, all configurations of the novel Bricks Game Task. In all three groups of participants, the administration order of the tasks was counterbalanced across the subjects.

### Statistical analysis

Pearson correlations (*r*) were used to identify any association between scores on the BGT task (in all configurations), and on the Digit Span task (both DSF and DSB) and Corsi Block-Tapping test. Finally, based on correlation analysis results, a Multiple linear regression analysis was run to reveal any associations between executive performance assessed with traditional working memory tasks (Digit Span task and Corsi Block-Tapping test) or with the novel BGT task. In the hypothesized model, we expected that M-BGT, CL-BGT, and CR-BGT tasks performance were predicted by scores on the Corsi Block-Tapping test, Digit Span task (DSF and DSB) and Age. To assess differences in group’s global performances, all dependent variables, obtained from the Corsi Block-Tapping test, Forward and Backward Digit Span task, and Matrix (M-BGT); Cluster (CL-BGT), and Cross (CR-BGT) BGT novel task span scores, were submitted to Multivariate analysis of variance (MANOVA), with Gender (M, F) and class Group (Gr1, Gr2, Gr3) as factors. *Post-hoc* analyses were performed afterward (Tukey’s honestly significant difference, HSD). In addition, to assess potential performance differences between the three configurations of the BGT task, a mixed-model analysis of variance (ANOVA) has been run on BGT span scores. Performances in M-BGT, CL-BGT, and CR-BGT tasks have been analysed, with Group as between-factor and Configuration (Matrix, Cluster, Cross) as within-factor. When needed, Bonferroni’s *post-hoc* multiple comparisons were carried out. Alpha level was fixed at ⩽0.05. All statistical analyses were performed using IBM SPSS Statistics for Macintosh, version 25.0 (IBM Corp., Armonk, NY, USA).

## Results

### Associations between traditional working memory tasks and BGT

Pearson correlations (*r*) results showed a significant positive correlation between M-BGT score and both CBT (*r* = 0.48, *p* < 0.001) test and DS-B (*r* = 0.36, *p* < 0.001). Regarding Cluster configuration, we found a positive correlation between CBT test (*r* = 0.42, *p* < 0.001) and both DS-B (*r* = 0.39, *p* < 0.001) and FW (*r* = 0.19, *p* = 0.003). Positive correlation was also detected between Cross configuration and CBT score (*r* = 0.5, *p* < 0.001), and both DS-B (*r* = 0.44, *p* < 0.001) and *F* (*r* = 0.2, *p* = 0.002).

As for the multiple linear regression, the results indicated that predictors explained 28% of the variance (*R*^2^ = 0.28, *F*_4,223_ = 21.05, *p* < 0.0001) of M-BGT score. It was found that both CBT performance (*β* = 0.37, *t* = 5.71, *p* < 0.000) and Age (*β* = 0.16, *t* = 2.5, *p* = 0.01) significantly predicted M-BGT score (*β* = 0.56, *p* < 0.001), indicating that both a higher age and a better CBT score predict better M-BGT test performance. Moreover, predictors explained 23% of the variance (*R*^2^ = 0.23, *F*_4,224_ = 16.74, *p* < 0.0001) of CL-BGT score. CBT (*β* = 0.27, *t* = 4.04, *p* < 0.0001), DS-F(*β* = 0.13, *t* = 2.22, *p* = 0.02) and DS-B (*β* = 0.22, *t* = 3.2, *p* = 0.002) significantly predicted CL-BGT performance, showing how higher scores result in a better CL-BGT performance. Finally, the predictors explained 35% of the CR-BGT score variance (*R*^2^ = 0.35, *F*_4,224_ = 29.95, *p* < 0.0001). CBT (*β* = 0.34, *t* = 5.44, *p* < 0.0001), DS-F (*β* = 0.14, *t* = 2.51, *p* = 0.01), DS-B (*β* = 0.17, *t* = 2.73, *p* = 0.007) and Age (*β* = 0.2, *t* = 3.42, *p* = 0.001) were positively associated with CR-BGT performance.

### Differences in global performances between groups

MANOVA showed the main significant effect for Group (*F*_12,434_ = 15.06; *p* < 0.0001; pη^2^ = 0.3; Wilks’ *Λ* = 0.5) indicating that Group 3 obtained better global executive performance (CBT = 4.9 ± 0.97; M-BGT = 5 ± 0.97; CL-BGT = 4.2 ± 0.8; CR-BGT = 4.83 ± 1.04; DS-FW = 4.67 ± 1.26; DS-BW = 4.15 ± 0.98) than both Group 2 (CBT = 4.03 ± 0.63; M-BGT = 4.4 ± 0.8; CL-BGT = 3.95 ± 0.61; CR-BGT = 4.2 ± 0.62; DS-F = 4.64 ± 0.8; DS-B = 3.08 ± 0.78) and Group 1 (CBT = 4.05 ± 0.7; M-BGT = 4.15 ± 0.66; CL-BGT = 3.93 ± 0.72; CR-BGT = 3.86 ± 0.71; DS-F = 4.5 ± 0.96; DS-BW = 2.64 ± 0.63). Post-hoc comparisons revealed a significant difference between Group 3 and both Group 1 and 2 in CBT (Gr1 vs Gr3 = *p* < 0.0001; Gr2 vs Gr3 = *p* < 0.0001), in M-BGT (Gr1 vs Gr3 = *p* < 0.0001; Gr2 vs Gr3 = *p* < 0.0001) and in CL-BGT (Gr1 vs Gr3 = *p* < 0.0001; Gr2 vs Gr3 = *p* < 0.0001). Significant differences were found also between Group 1 and 2; Group 1 and 3; Group 2 and 3 in CR-BGT (Gr1 vs Gr2 = *p* = 0.04; Gr1 vs Gr3 = *p* < 0.0001; Gr2 vs Gr3 = *p* < 0.0001) and DS-BW (Gr1 vs Gr2 = *p* = 0.003; Gr1 vs Gr3 = *p* < 0.0001; Gr2 vs Gr3 = *p* < 0.0001). No other significant main or interaction effects were observed.

### Differences between BGT configurations

**Fig. 2:**
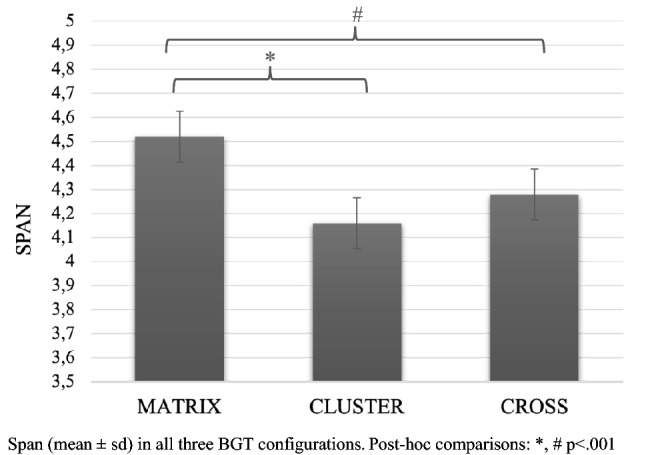
Span (mean ± sd) in all three BGT configurations. Post-hoc comparisons: *, # *p* < .001

**Fig. 3:**
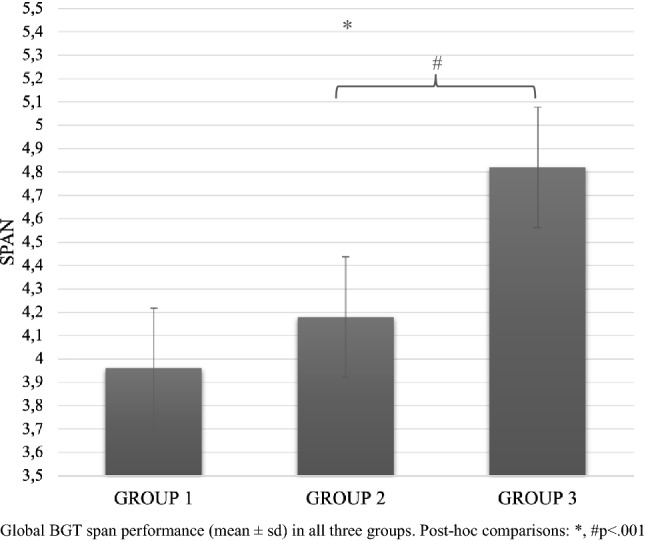
Global BGT span performance (mean ± sd) in all three groups. Post-hoc comparisons: *, #*p* < .001

Mixed-model ANOVA showed the main significant effect for Configuration (*F*_2,458_ = 19.19; *p* < 0.001; *pη*^2^ = 0.07), revealing better performance (i.e., longer span) in Matrix configuration (4.52 ± 0.92) to both Cluster (4.16 ± 0.05) and Cross (4.28 ± 0.05). Bonferroni’s multiple comparisons revealed a significant difference between Matrix and both Cluster (*p* < 0.001) and Cross (*p* < 0.001) configurations (Fig. 2). As expected, the main effect for Group was detected (*F*2,229 = 44.55; *p* < 0.001; *pη*^2^ = 0.3), indicating that grade level had a significant impact on performance (Gr1 = 3.96 ± 0.06; Gr2 = 4.18 ± 0.08; Gr3 = 4.82 ± 0.07; Fig. 3). Bonferroni’s multiple comparisons revealed a significant difference between Group 3 and both Group 1 (*p* < 0.001) and Group 2 (*p* < 0.001). No significant interaction effects were observed.

## Discussion

In this study, a new task designed to measure children’s visuospatial abilities was validated. To examine how children represent and manipulate space, we developed an assessment tool (i.e., the Bricks Game Task, BGT) that is rather more ecologically valid than classic span tasks. To confirm existing evidence that spatial skills evolve in parallel with neural development, we administered the new Bricks Game Task (BGT) to children of different age and education levels. In line with our hypotheses, we found significant differences in the performance of children from different groups based on their educational level. In the new BGT, as well as in the classic DS and CBT, the level of education had a significant influence on the performance of the children, allowing those from higher primary years to have higher spans than those from lower primary years. This result is in line with a large body of literature associating visuospatial abilities with academic success (Lambert & Spinath, 2018; Serra et al., 2021; Tikhomirova et al., 2020). A variety of studies had stated that visuospatial skills predict adolescents’ and children’s achievements and general academic performance in early elementary school grades (Kyttälä & Lehto, 2008) as well as their mathematical (Li & Geary, 2017) and reading (Pham & Hasson, 2014) abilities. Furthermore, the improvement in visuospatial abilities with advancing age has to do with neural development mechanisms (Alejandre-Gomez et al., 2007; Giedd et al., 1999; Méndez-López et al., 2009; Vuontela et al., 2003). Several studies on classic CBT have shown that children’s performance increases with age. This is clearly illustrated in a study by Logie (2003) in which recall and recognition versions of both CBT and visual pattern tasks were administered to children of 5/6, 8/9 and 11/12 years of age. In a study of CBT with 288 children between the ages of 7 and 15 years, Isaacs and Vargha-Khadem (1989) found that span increased from an average of 4.1 to 5.6 blocks. Among the most influential neurodevelopmental processes, involved in visuospatial abilities, is surely the maturation of functions associated with the central executive component of working memory. A range of cognitive functions are associated with the central executive, including attention, inhibition, switching, planning, and simultaneous storage and processing of information (see Baddeley, 2000). Pickering (2001) contends that in older children and adults, the efficient allocation of (phonological and visuospatial) slave system resources by the central executive is critical to the operation of working memory. Luciana and Nelson (1998) also suggest that early in the process of developing working memory, fundamental perception and motor functions are refined, but afterward, neural networks mature, thus integrating complex processes associated with multiple cognitive domains. It follows that the maturation of the neural system, and corresponding changes in cognitive functioning, play an important role in the development of visuospatial abilities.

In our study, we decided to administer the BGT in three different spatial configurations to evaluate also how much the organization of the nearby space influences the memory processes. The use of three different spatial configurations is one of the innovative aspects that characterise the BGT. Compared to Cross and Cluster, children performed significantly better in the Matrix configuration, regardless of education level. A similar attempt to study visuospatial abilities using different spatial configurations was made by Sorrentino et al., (2019). In their study, the administered a spatial task in which the child was free to move, adopting exploratory behaviors in accordance with the environment. Children were asked to explore an open space to search for nine white bricks arranged in three spatial configurations: Cross, Cluster and Matrix. Their findings revealed that Cross configuration was the hardest one to explore, since the best strategy is not immediately suggested by the geometry, and it requests further cognitive abilities, such as cognitive flexibility. Cluster configuration was easier to explore since it offers the possibility to use a chunking strategy, which is visiting clusters within a cluster before moving to another cluster (Murdock, 1995, 2005; Schyns et al., 1998). This strategy implies a hierarchical organization of memory, reducing the working memory load, and improving the overall performance (Cohen et al., 2003; Terrace & McGonigle, 1994). The Matrix configuration was the easiest to explore. In previous studies, Foti et al., (2011, 2015) reported that pre-schoolers explored the Matrix configuration using a structured search pattern characterised by the shortest transitions between compartments, and that these children were able to successfully navigate an open environment as early as age 6 when these structured patterns were present. Considering all this evidence, it would appear that the spatial strategy used to explore the Matrix configuration is the easiest one to learn and is developed already at a very young age.

As a final step in our validation study, we investigated the association between children's performance on the BGT and performance on classical span tasks like the CBT and the DS. We found a strong association between the outcomes of BGT and those of CBT and DS. This result not only makes BGT valid and reliable but also gives us the possibility to use a more ecological tool to study visuospatial abilities in children (Fanuel et al., 2020). Although CBT and DS are widely used and considered reliable tools for the study of children's cognitive functioning, they are not fully specific for visuospatial abilities and lack ecological validity. In recent years, there has been a growing need to develop neuropsychological assessment tools that are ecologically valid in children. The use of task that are able to overcome the limitations of pen-and-paper testing in hospital and laboratory environment, would make it easier to draw conclusions about the functionality of children's adaptation and development (Olson et al., 2013; Price et al., 2003; Spencer-Smith et al., 2020). With this in mind, BGT appears as a sensitive visuospatial working memory task that can be used to assess children’s executive performance in an ecological way.

## Future directions

The use of a tool like the Bricks Game Task in both clinical and experimental settings gives us many advantages. Among them, there is the possibility of testing visuospatial working memory skills in an ecological context. In addition, allowing us to gain a better understanding of the visuospatial skills of children, BGT is also easily distributable in school and educational contexts, where detecting cognitive deficiencies at an early age, is crucial for children’s education and development. BGT is also an assessment tool that can be adapted for digitization. An interesting future outcome of this research would be the conversion of the BGT into a non-immersive virtual reality (VR) test for analysing visuospatial abilities (Korečko et al., 2018). Due to digitization, developmental pathways are changing, and children are adapting to new stimuli from the environment. Therefore, diagnostic screenings or research methods must also adapt, creating paradigms that are not constrained by laboratory conditions and that more closely represent reality as it applies to children. Creating a digitized version of the BGT would allow us to meet the growing demand for assessment tools that are more practical and ecologically valid. Bringing spatial cognition analysis into the virtual world, researchers have already created virtual versions of the main large-scale behavioural tasks frequently used in spatial cognition such as Morris Water Maze (MWM) and Radial Arm Maze (RAM) (Bohbot et al., 2004; Cornwell et al., 2008; Iaria et al., 2003; Palombi et al., 2022; Patel et al., 2022), although there they are still few fully immersive versions (León et al., 2018; Somma et al., 2021), These digitized versions provide several advantages, including the convenience of sharing tasks among several research groups, and the possibility of easily entering the data obtained into scientific databases (Palombi et al., 2022). The digitization of the BGT, by using a non-immersive VR system, it will make possible to create a 2D virtual environment of the three configurations projected onto a computer screen, where the children use a PC monitor, keyboard, and mouse, as well as a gamepad or joystick to perform the task (Marková et al., 2015). Thus, the BGT will be presented to the child as a game, and this will increase his motivation to complete it, enabling the experimenter to make a more comprehensive, accurate and realistic evaluation assessment of his/her spatial working memory abilities. Moreover, we can say in conclusion that digitizing the BGT, as with the large-scale spatial ability tests already in place, has many clinical and scientific advantages, as it provides us with tools that allow us to respond to the growing demand for a more ecological and more effective assessment tool for children.

## Data Availability

The data that support the findings of this study are available from the corresponding author upon request.
